# Efficacy of tripterygium glycosides combined with ARB on diabetic nephropathy: a meta-analysis

**DOI:** 10.1042/BSR20202391

**Published:** 2020-11-13

**Authors:** Xue Wu, Youye Huang, Yao Zhang, Chunling He, Yongli Zhao, Lizhuo Wang, Jialin Gao

**Affiliations:** 1 Department of Endocrinology and Genetic Metabolism, The First Affiliated Hospital of Wannan Medical College (Yijishan Hospital of Wannan Medical College), Wuhu 241002, China; 2 Institute of Endocrine and Metabolic Diseases, Yijishan Hospital of Wannan Medical College, Wuhu 241002, China; 3 Anhui Province Key Laboratory of Biological Macro-molecules Research (Wannan Medical College), Wuhu, China; 4 Department of Biochemistry, Wannan Medical College, Wuhu, China

**Keywords:** angiotensin II receptor blocke, diabetic nephropathy, tripterygium glycosides

## Abstract

The purpose of this meta-analysis was to evaluate the beneficial and adverse effects of tripterygium glycosides (TGs) combined with angiotensin II receptor blocker (ARB) on diabetic nephropathy (DN). We searched for randomized controlled trials (RCTs) in PubMed, Embase, Cochrane Central Register of Controlled Trials, Web of Science, China National Knowledge Infrastructure (CNKI), Wanfang Data, Chinese Biomedical Literature Database, China Science and Technology Journal Database up to June 2017. Weighted mean difference (WMD) and standardized mean difference (SMD) were used for continuous variables and all variables were expressed by 95% confidence interval (CI). Twenty-three studies with 1810 DN patients were included in this meta-analysis. TG combined with ARB statistically significantly improved 24-h urinary total protein (24-h UTP) (SMD = −1.46; 95% CI = −1.84 to −1.09; *P*<0.00001), urinary albumin excretion rate (UAER) (SMD = −6.9; 95% CI = −9.65 to −4.14, *P*<0.00001), serum creatinine (SCr) (WMD = −7.65.14; 95% CI = −12.99 to −2.31; *P*=0.005) and albumin (Alb) (WMD = 5.7; 95% CI = 4.44 to 6.96; *P*<0.00001) more than did ARB alone. TG combined with ARB statistically significantly affected the level of serum glutamic pyruvic transaminase (SGPT) (WMD = 1.08; 95% CI = 0.04 to 2.12, *P*=0.04) more than did ARB alone. Compared with ARB alone, TG combined with ARB showed no significant difference in improving blood urea nitrogen (BUN) and hemoglobin A1c (HbA1c). Minor side effects from the combined treatment were observed and mainly focused on the abnormal liver function. TG combined with ARB offers a novel concept in treating DN, more high-quality RCTs are needed for better understanding and applying the combined treatment in DN.

## Introduction

Diabetes mellitus (DM) is a common metabolic disease worldwide that has rapidly increased in prevalence. It has been reported that the global diabetes population will reach 592 million by 2035 [1]. 2010 data showed that the prevalence of diabetes and prediabetes was 11.6 and 50.1% in a sample of Chinese adults. This means that approximately 113.9 and 493.4 million Chinese adults may suffer from diabetes and prediabetes, respectively [2]. Diabetic nephropathy (DN), as a microvascular complication of diabetes, is characterized by microalbuminuria, glomerular basement membrane thickening, mesangial matrix expansion and tubulointerstitial fibrosis [3]. DN is incurable and often leads to end-stage renal disease (ESRD). However, the pathogenesis of diabetes is still not clear and there are currently no effective treatments to prevent the progression to ESRD. Currently, clinical treatment is mainly focused on the control of blood pressure, blood glucose and inhibition of the renin–angiotensin system (RAS) [4]. Angiotensin-converting enzyme inhibitors (ACEI) and angiotensin II receptor blocker (ARB) are blockers of RAS [5]. In cases of DN, combined treatment using ACEI and ARB is more successful in decreasing 24-h proteinuria than ACEI alone [6]. The American Diabetes Association (ADA) recommends ARB for type 2 DN [7].

As a traditional Chinese medicine (TCM), tripterygium glycosides (TGs) have attracted attention in the treatment of DN. TG is a fat-soluble substance isolated from the core of roots of tripterygium which has immunosuppressive and anti-inflammatory effects. TG is often used to treat rheumatoid arthritis, chronic kidney disease and other autoimmune diseases [8,9]. Moreover, TG has been used to reduce proteinuria and protect the kidney for more than 20 years [10].

Therefore, we collected randomized controlled trials (RCTs) and used meta-analysis to compare beneficial and adverse effects of using the combination of TG and ARB with ARB alone for treating DN.

## Materials and methods

### Database and search strategies

We searched PubMed, Embase, Cochrane Central Register of Controlled Trials, Web of Science, China National Knowledge Infrastructure (CNKI), Wanfang Data, Chinese Biomedical Literature Database (CBM), China Science and Technology Journal Database (VIP) up to June 2017. The following keywords and Medical Subject Headings (MeSH) were searched: diabetic nephropathies, diabetic nephropathy, diabetic kidney disease, diabetic kidney diseases, DN and TGs.

### Inclusion and exclusion criteria

Inclusion criteria: (1) Research type: RCT design. (2) Research subjects: patients were clinically diagnosed with DN. (3) Interventions: The control group was treated with ARB and the experimental group was added with TG on the basis of the control group. Other measures were consistent in the two groups. (4) Outcome measures: 24-h urinary total protein (24-h UTP), urinary albumin excretion rate (UAER), serum creatinine (SCr), blood urea nitrogen (BUN), albumin (Alb), hemoglobin A1c (HbA1c) and serum glutamic pyruvic transaminase (SGPT).

Exclusion criteria: (1) Non-randomized controlled or semi-randomized controlled trials. (2) Other specific types of diabetes other than type 1 DM (T1DM) and type 2 DM (T2DM). (3) Kidney damage resulting from diseases other than T1DM or T2DM. (4) Other Chinese medicines being used in the control group or the experimental group. (5) Lack of rigorous experimental design, inappropriate statistical method or lack of related outcome measures.

### Data extraction

Two researchers screened the studies and extracted data independently according to the specified criteria and differences between researchers were resolved by discussion. If two researchers failed to reach a consensus, a third researcher would decide. The following data were extracted: basic information of studies (authors, title of the study, publication year, study design, sample sizes), characteristics of patients (age, sex, duration of diabetes), details of interventions (dosage, frequency, treatment duration), outcome measures (24-h UTP, UAER, SCr, BUN, Alb, HbA1c, SGPT) and adverse effects. Extracted data were given as mean ± standard deviation (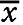 ± *s*).

### Quality assessment

We evaluated the quality of studies using the Cochrane Collaboration’s tool for assessing risk of bias. The tool includes the following seven sections: random sequence generation (selection bias), allocation concealment (selection bias), blinding of participants and personnel (performance bias), blinding of outcome assessment (detection bias), incomplete outcome data (attrition bias), selective reporting (reporting bias) and other biases.

### Statistical analysis

We used the Review Manager 5.3 software for statistical analysis. Weighted mean difference (WMD) or standardized mean difference (SMD) were used for continuous variables, and odds ratio (OR) was used for dichotomous variables, all variables were expressed by 95% confidence interval (CI). We used *I^2^* statistic to assess heterogeneity of included RCTs. If *I^2^* was lower than 50%, the heterogeneity was considered acceptable and we used a fixed-effects model to analyze data. If *I^2^* was higher than 50%, we used subgroup analysis or sensitivity analysis to deal with heterogeneity. If heterogeneity remained higher than 50%, we used a random-effects model to analyze data.

## Results

### Study search results and study characteristics

**Figure 1:**
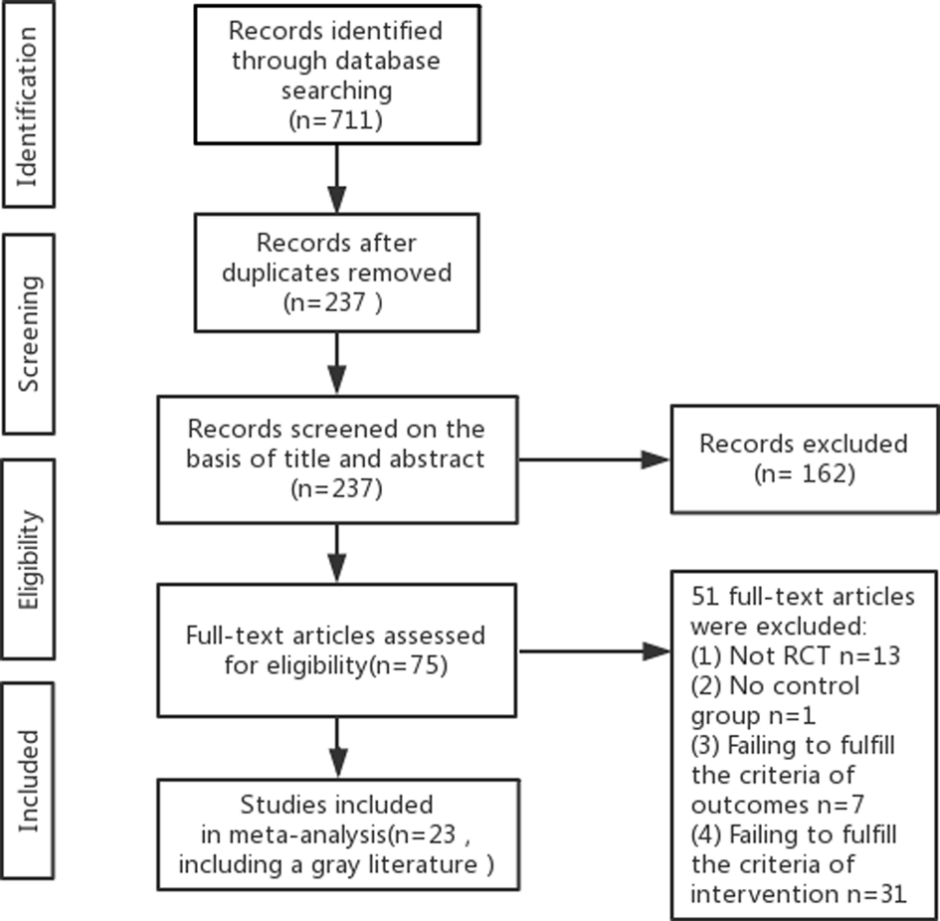
Flow diagram of study selection

Initially, 711 studies were screened out by nine databases. After removing duplicates and reading the title and abstract, 75 studies remained. From these 75 studies, we selected and read 23 RCTs in full to use for the meta-analysis. We included a dissertation [11] because its experimental design is reasonable and its data are complete (Figure 1).

A total of 1810 DN patients were included in the meta-analysis. Of these 1810 patients, 924 were in the experimental group and the remaining 886 were in the control group. The experimental groups were treated with a combination of TG and ARB and the control groups were treated with ARB alone. The duration of the treatments ranged from 2 to 48 weeks. In all the included studies, there was no significant difference in baseline data (age, sex, duration of diabetes) between the experimental group and control group in Table 1. The baseline characteristics of the included studies are summarized in Table 2.

**Table 1:** Comparison of baseline data

Variables	Heterogeneity		Model	Summary statistic	Effect value		
	*I^2^* (%)	*P*			Effect [95% CI]	Z	*P*
Gender	0	1.00	Fix	OR	1.12 [0.92, 1.36]	1.13	0.26
Age	0	0.47	Fix	MD	0.54 [−0.07, 1.15]	1.74	0.08
Duration	0	0.86	Fix	SMD	0.04 [−0.08, 0.16]	0.69	0.49

*P*<0.05, there was a statistical significance. Abbreviation: Fix, fixed-effects model.

**Table 2:** Baseline characteristics of the 23 studies

Study	Study’s design	Sample sizes		Treatment		Outcomes	Course (weeks)
		Experimental	Control	Experimental	Control		
Zhou 2013 [12]	RCT	15	15	TG 40 mg tid + Control group treatment	Valsartan 80–160 mg/d	24-h UTP, SCr, Alb, SGPT	8
Zhao 2011 [13]	RCT	23	23	TG 20 mg tid+Control group treatment	Valsartan160 mg qid	24-h UTP, SCr, Alb	12
Zhang 2012 [14]	RCT	50	50	1–12 weeks: TG 20 mg tid 13–24 weeks: TG 10 mg tid + Control group treatment	Irbesartan 75 mg qid–150 mg bid	24-h UTP, SCr, Alb	24
Zhang 2015 [11]	RCT	20	20	TG 1 mg/kg·d + Control group treatment	Valsartan160 mg qid	SCr, SGPT BUN, HbA1c, Alb	12
Zhang 2015 [15]	RCT	66	65	TG 30 mg bid + Control group treatment	Valsartan 80 mg qid	24-h UTP	48
Ye 2016 [16]	RCT	105	105	TG 20 mg tid + Control group treatment	Valsartan 80 mg qid	UAER, SCr	12
Yang 2016 [17]	RCT	14	16	TG 1 mg/kg·d tid + Control group treatment	Losartan 50 mg qid	24-h UTP	8
Wu 2010 [18]	RCT	32	30	TG 40 mg tid + Control group treatment	Telmisartan 80 mg bid	24-h UTP, UAER, SCr, Alb, HbA1c, SGPT	12
Wang 2013 [19]	RCT	32	30	1–8 weeks: TG 20 mg tid 9–24 weeks: TG 10 mg tid + Control group treatment	Telmisartan 40–80 mg/d	24-h UTP, SCr, BUN, HbA1c	24
Wang 2016 [20]	RCT	26	26	TG 0.3–0.5 mg/kg tid + Control group treatment	Valsartan 80 mg qid	24-h UTP	12
Wang 2012 [21]	RCT	52	30	1–8 weeks: TG 20 mg tid 9–24weeks: TG 20 mg qid + Control group treatment	Valsartan 160 mg qid	24-h UTP, UAER, SCr, Alb, HbA1c, SGPT	24
Tan 2010 [22]	RCT	25	23	1–12 weeks: TG 20 mg tid 13–24 weeks: TG 10 mg tid + Control group treatment	Irbesartan 75 mg qid–150 mg bid	24-h UTP	24
Shen 2014 [23]	RCT	90	90	TG 20 mg tid + Control group treatment	Irbesartan 150 mg bid	24-h UTP	2
Bareti 2011 [24]	RCT	43	42	1–4 weeks: TG 20 mg tid 5–12 weeks: reduce dosage by 10% per week to 10–20 mg, tid + Control group treatment	Valsartan 80–160 mg qid	24-h UTP	12
Liu 2015 [25]	RCT	30	30	TG 1 mg/kg.d + Control group treatment	Irbesartan 300 mg bid	SCr	12
Li 2013 [26]	RCT	43	43	TG 20 mg tid + Control group treatment	Valsartan 80 mg qid	UAER, SCr, Alb	36
Li 2011 [27]	RCT	20	20	TG 20 mg tid + Control group treatment	Valsartan	24-h UTP, BUN, Alb, SGPT	14
Li 2014 [28]	RCT	48	48	1–10 weeks: TG 20 mg tid 11–24 weeks: TG 10 mg tid + Control group treatment	Irbesartan 75 mg qid–150 mg bid	24-h UTP, Alb	24
Jiang 2015 [29]	RCT	62	64	TG 20 mg tid + Control group treatment	Telmisartan 80 mg qid	24-h UTP, BUN, HbA1c	12
Huang 2010 [30]	RCT	25	23	1–12 weeks: TG 20 mg tid 13–24 weeks: TG 10 mg tid + Control group treatment	Irbesartan 75 mg qid–150mg bid	24-h UTP	24
He 2010 [31]	RCT	31	29	1–8 weeks: TG 20 mg tid 9–24 weeks: TG 10 mg tid + Control group treatment	Valsartan 80–160 mg/d	24-h UTP, UAER, SCr, HbA1c, Alb	24
Tong 2012 [32]	RCT	40	35	TG 20 mg tid + Control group treatment	Losartan100 mg/d	SCr, BUN, Alb	12
Cai 2012 [33]	RCT	32	29	TG 20 mg tid + Control group treatment	Valsartan 40–80 mg qid	24-h UTP, Alb	24

### Quality of included studies

**Figure 2:**
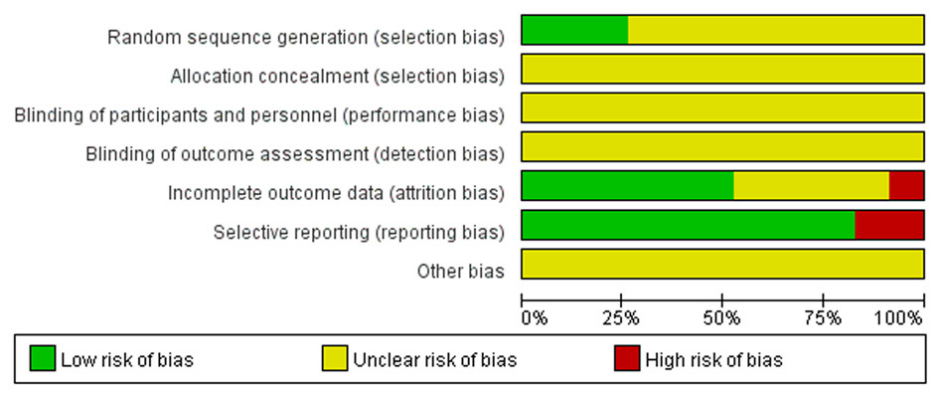
Risk of bias graph: each risk of bias item presented as percentages across included 23 studies

**Figure 3:**
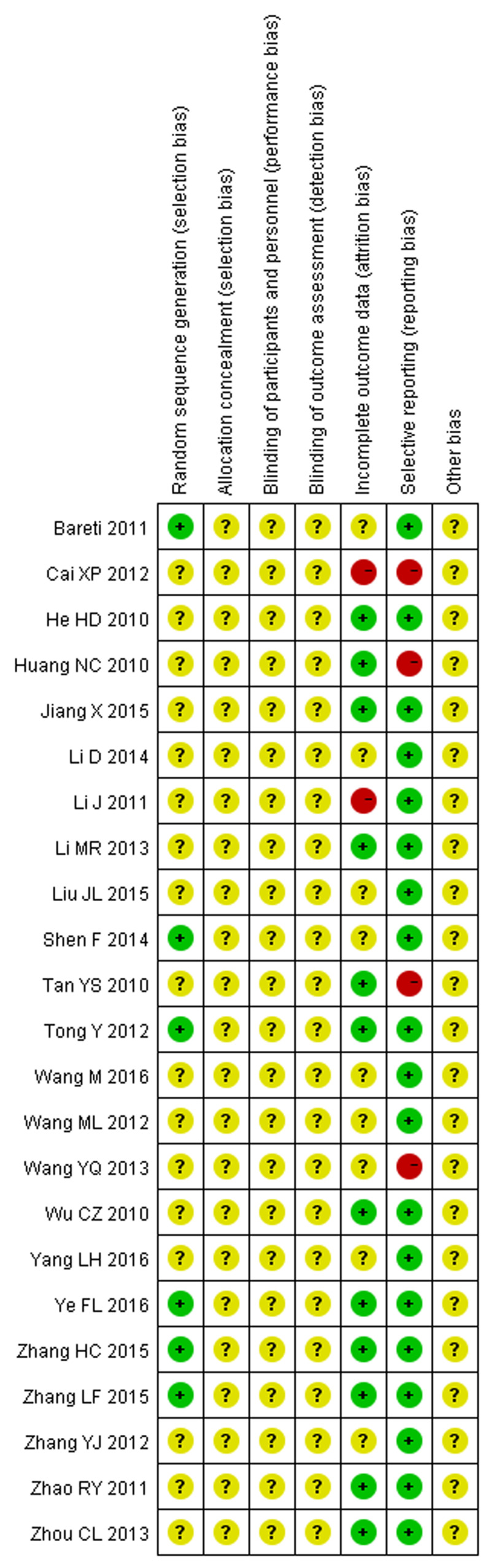
Risk of bias summary: each risk of bias item for included 23 studies

The quality of included studies was assessed using the Cochrane Collaboration’s tool for assessing risk of bias. There were only five studies [11,15,16,23,24] mentioned using random number table to group, most studies did not mention specific random methods. Only one study [14] referred to the envelope method, but did not describe the details, such as seal and light. Three studies [15,27,33] reported dropouts, failure to follow-up and elimination. Four studies [19,22,30,33] did not report complete outcomes and mentioned that bias existed. None of the studies discussed other bias (Figures 2 and 3).

### Quantitative data analysis

#### Effect on 24-h UTP

**Figure 4:**
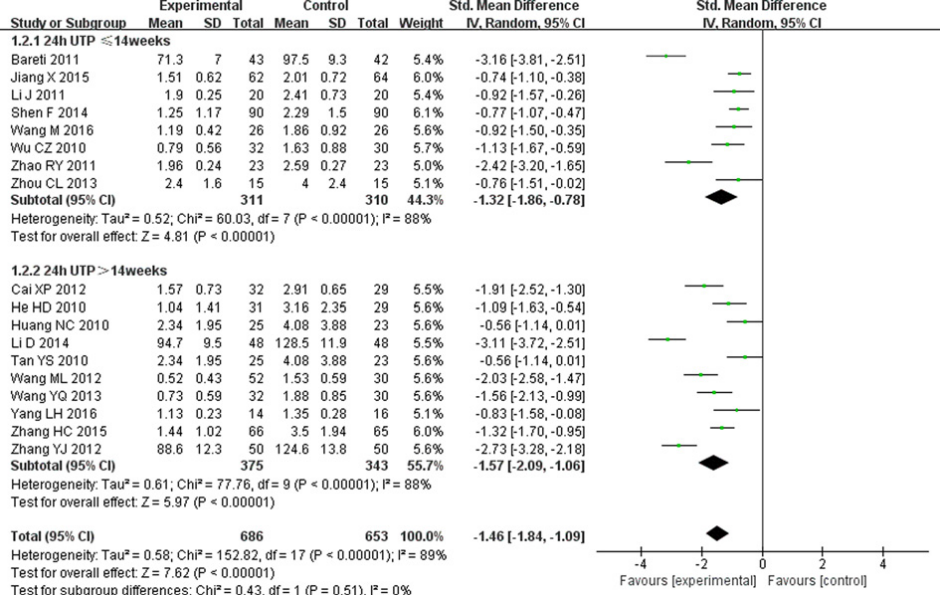
Forest plots of 24-h UTP after treatment in DN patients

Mass proteinuria is one of the diagnostic criteria for DN. Eighteen studies [12–15,17–24,27–31,33] reported changes in 24-h UTP. Considering the significant heterogeneity (*P*<0.00001, *I^2^* = 89%), random-effects model was used. The result of meta-analysis showed that TG combined with ARB is more effective at reducing 24-h UTP (SMD = –1.46; 95% CI = −1.84 to −1.09; *P*<0.00001) than ARB alone. Subgroup analysis also showed that there was a statistically significant difference in 24-h UTP between the experimental group and control group, whether the treatment lasted less than 14 weeks (SMD = −1.32; 95% CI = −1.86 to −0.78; *P*<0.00001) or more than 14 weeks (SMD = −1.57; 95% CI = −2.09 to −1.06; *P*<0.00001) (Figure 4).

#### Effect on UAER

**Figure 5:**
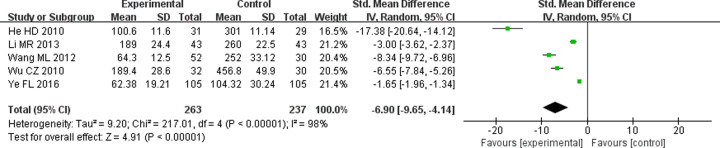
Forest plots of UAER after treatment in DN patients

Microalbuminuria was evaluated as an early clinical manifestation of DN using UAER. Because five studies [16,18,21,26,31] exhibited significant heterogeneity (*P*<0.00001, *I^2^* = 98%), random-effects model was used. The result showed that TG combined with ARB could improve UAER better than ARB alone (SMD = −6.9; 95% CI = −9.65 to −4.14, *P*<0.00001) (Figure 5).

#### Effect on SCr

**Figure 6:**
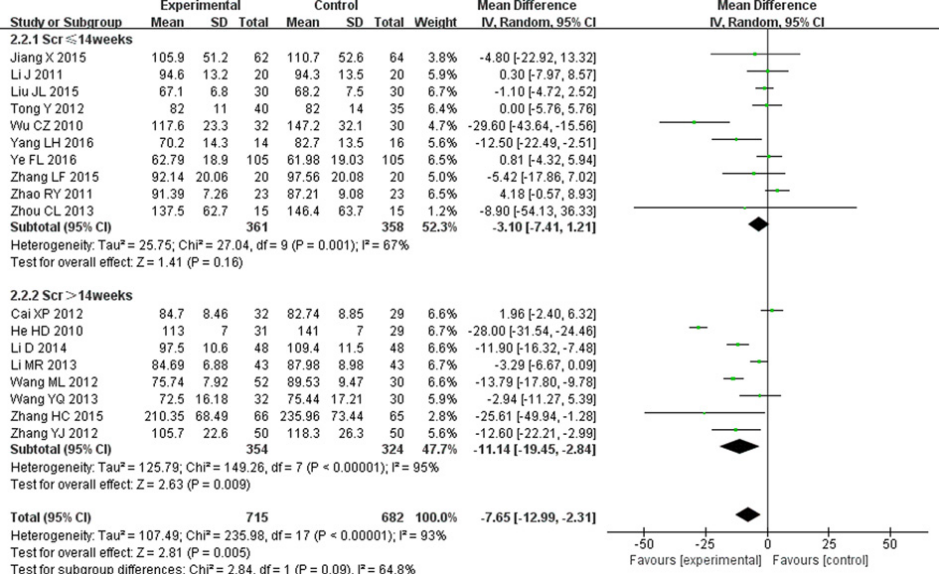
Forest plots of SCr after treatment in DN patients

SCr as the main indicator of kidney function plays an important role in the diagnosis and prognosis evaluation of DN [34]. Eighteen studies [11–19,21,25–29,31–33] mentioned the effect of combined treatment on SCr. Significant heterogeneity (*P*<0.00001, *I^2^* = 93%) was identified and analyzed the data by random-effects model. Meta-analysis showed that TG combined with ARB could improve SCr better than ARB alone (WMD = −7.65; 95% CI = −12.99 to −2.31; *P*=0.005) in DN patients. Subgroup analysis showed there was no difference in SCr between the experimental group and control group if treatment lasted less than 14 weeks (WMD = −3.1; 95% CI = −7.41 to −1.21; *P*=0.16), but there was a significant difference in SCr between the experimental group and control group if treatment lasted more than 14 weeks (WMD = −11.14; 95% CI = −19.45 to −2.84; *P*=0.009) (Figure 6).

#### Effect on BUN

**Figure 7:**
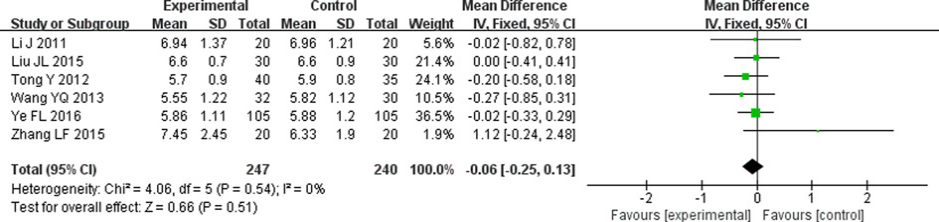
Forest plots of BUN after treatment in DN patients

BUN also is an indicator of kidney function, often used to reflect the change in kidney function in DN patients [35]. The effect on BUN was mentioned in seven studies [11,16,19,25,27,29,32] and significant heterogeneity was found across these studies (*P*=0.0005, *I^2^* = 75%). We conducted a sensitivity analysis by deleting a study [29] to eliminate heterogeneity (*P*=0.54, *I^2^* = 0) and analyzed data with fixed-effects model. The result indicated that there was no significant difference in reducing BUN between TG combined with ARB and ARB alone in DN patients (WMD = −0.06; 95% CI = −0.25 to −0.13; *P*=0.51) (Figure 7).

#### Effect on Alb

**Figure 8:**
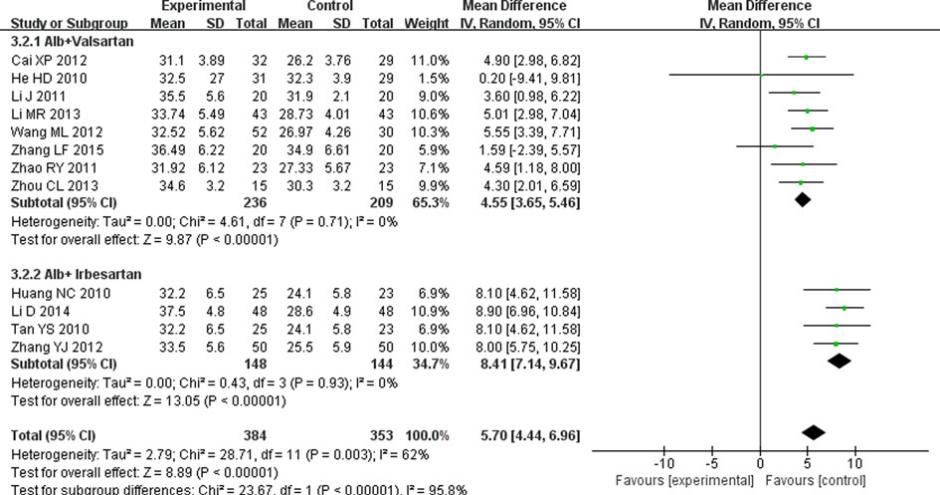
Forest plots of Alb after treatment in DN patients

The serum alb concentration is related to glomerular filtration rate [36], and its levels decline in DN patients. Twelve RCTs [11–14,21,22,26–28,30,31,33] significant heterogeneity were eligible to analyze (*P*=0.003, *I^2^* = 62%). Result conducted by random-effects model showed ARB combined with TG increased Alb more significantly compared with only ARB use (WMD = 5.7; 95% CI = 4.44 to 6.96; *P*<0.00001). Furthermore, whatever TG combined with valsartan (WMD = 4.55; 95% CI = 3.65 to 5.46; *P*<0.00001) or irbesartan (WMD = 8.41; 95% CI = 7.14 to 9.67; *P*<0.00001), they all could effectively improve Alb (Figure 8).

#### Effect on HbA1c

**Figure 9:**
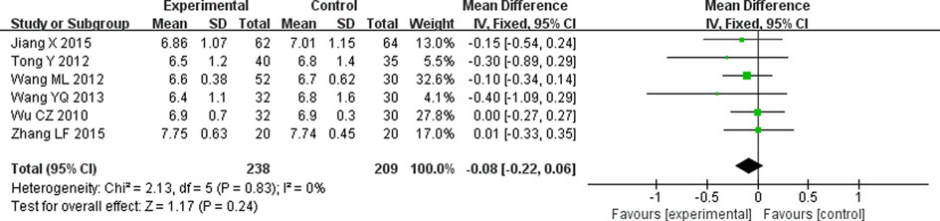
Forest plots of HbA1c after treatment in DN patients

HbA1c is used to monitor average blood sugar levels over 2–3 months in DN patients. Seven RCTs [11,18,19,21,29,31,32] were identified for the effect of TG combined with ARB on HbA1c. The heterogeneity changed from 79 to 0% after a sensitivity analysis by deleting a study [32]. The result performed by a fixed-effects model showed TG combined with ARB had no advantage in regulating HbA1c compared with ARB alone (WMD = −0.08; 95% CI = −0.22 to 0.06; *P*=0.24) (Figure 9).

#### Effect on SGPT

**Figure 10:**
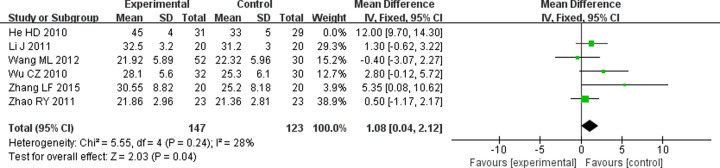
Forest plots of SGPT after treatment in DN patients

SGPT is an important liver enzyme, and its levels can reflect the changes of liver function after treatment in DN patients. Five RCTs [11,13,18,21,27] mentioned the effect of treatments on SGPT. Fixed-effects model was applied since the mild heterogeneity (*P*=0.24, I^2^ = 28%). Meta-analysis showed that the negative effect on SGPT was more obvious in TG combined with ARB than ARB alone (WMD = 1.08; 95% CI = 0.04 to 2.12; *P*=0.04) (Figure 10).

### Publication bias

**Figure 11:**
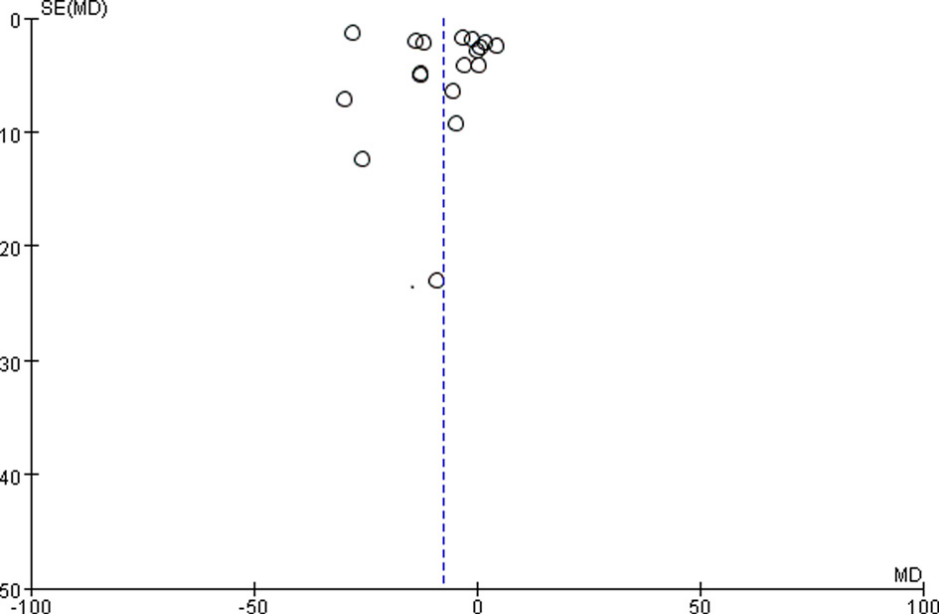
Funnel plot of publication bias for the effects of TG with ARB on DN

We used 18 studies [11–19,21,25–29,31–33] that mentioned SCr to make funnel plot and assessed their publication bias. The data are concentrated in the upper part of the funnel plot. This indicates that most of the studies are large samples. There was publication bias because this funnel plot is asymmetrical. In this meta-analysis, the heterogeneity across the studies and low quality of research methods might be related to funnel plot asymmetry (Figure 11).

### Adverse effects

Fourteen studies [11–13,15,16,18,21,22,26,27,30–33] mentioned adverse effects, eight studies [14,17,19,20,23–25,28] did not mention adverse effects and one study [29] mentioned no adverse effects occurred. Thirty-four patients observed abnormal liver function, including 30 patients in the experimental group with an incidence of 3.2%, and 4 patients who were not clearly grouped. And in the experimental group, ten patients observed gastrointestinal symptoms with an incidence of 1.1%, and four patients observed reduction in WBC with an incidence of 0.4%. Four patients who were not clearly grouped observed menstrual disorder with an incidence of 0.2%. Other adverse effects such as infection or deterioration of kidney function were not mentioned in the all studies. Most of the patients who had adverse effects could be improved after symptomatic treatment, except for one patient withdrawal.

## Discussion

There are still no effective treatments for DN or ESRD. ARB can improve proteinuria both in early and late stages of type 2 DN [7] and alleviate the progression of kidney injury. TG is widely used to treat primary glomerulonephritis and immune-related nephritis. In DN, TG not only improved proteinuria, but also alleviated kidney pathological changes and reduced the inflammation levels of kidney via p38 mapk pathway [37,38]. In order to research the effect of combined TG and ARB on DN, we performed this meta-analysis. We demonstrated that the effect of TG with ARB was better for 24-h UTP (*P*<0.05), UAER (*P*<0.05), SCr (*P*<0.05) and Alb (*P*<0.05) when compared with the control group of using an ARB alone in patients with DN. However, combination treatment had no obvious advantage in improving BUN (*P*>0.05) and HbA1c (*P*>0.05). We can speculate that compared with ARB alone, TG combined with ARB can repair the renal function of DN patients to a certain extent, but has no significant improvement in regulating blood sugar. The incidence of adverse effects was low and most of the adverse effects were observed in the experimental group. Abnormal liver function was the main adverse effect, and the effect of TG combined with ARB on liver enzyme is more obvious than that of ARB alone. Although liver injury can be improved in the majority of patients by treating, this is still worth noting in clinical treatment.

Unfortunately, there were limitations in this meta-analysis. (1) This meta-analysis lacks high-quality RCTs, most did not describe specific randomization, allocation concealment and binding. In addition, some studies did not show the loss of follow-up and the outcomes of a few studies were also incomplete. These are most likely to cause selection bias, performance bias, detection bias, attrition bias and reporting bias (2). The diagnostic criteria for DN patients is not uniform and this may cause the included patients to be at different stages of DN. (3) The studies included are all Chinese and some outcomes contained only a smattering of studies. (4) The outcome measures observed were not comprehensive. For example, blood pressure is an important outcome measure, because the antihypertensive effect of ARB can not only reduce the incidence of kidney disease but also benefit the cardiovascular system [39]. Unfortunately, blood pressure changes in patients were not recorded in the included studies. (1), (2) and (3) may be related to the high heterogeneity of some outcome measures. Heterogeneity was still present, even if we performed subgroup analysis and sensitivity analysis to find the source of heterogeneity. We did not give up these results for 24-h UTP, UAER and SCr, even though they had heterogeneity. This is because we believed that these analytical results were needed to complement high-quality RCTs and could provide some references for clinical treatment. These results are also interesting. For example, TG combined with ARB could improve 24-h UTP in patients regardless of the length of treatment while the effect on improving SCr mainly appeared in patients with a longer course of treatment. Besides, the stable results on BUN, Alb, HbA1c, SGPT were also produced. The negative results on BUN and HbA1c were conducted only by a small amount of studies and still lacked high-quality RCTs to support or even deny, although their heterogeneities have been eliminated and they have achieved the stability of data.

TCM has a long history of medicinal use in China and the side effects of TCM are relatively minor. The combination of TCM and Western medicine could prove to be an important method for treating DN. This meta-analysis provides a new perspective in regard to the combined treatment using TG and ARB on DN. For better understanding and optimizing TG combined with ARB in DN, we need to standardize clinical trials and analyze larger multicenter, random and blind RCTs.

## Abbreviations

ACEI: angiotensin-converting enzyme inhibitor
Alb: albumin
ARB: angiotensin II receptor blocker
BUN: blood urea nitrogen
CI: confidence interval
DM: diabetes mellitus
DN: diabetic nephropathy
ESRD: end-stage renal disease
HbA1c: hemoglobin A1c
OR: odds ratio
RAS: renin–angiotensin system
RCT: randomized controlled trial
SCr: serum creatinine
SGPT: serum glutamic pyruvic transaminase
SMD: standardized mean difference
TCM: traditional Chinese medicine
TG: tripterygium glycoside
T1DM: type 1 DM
T2DM: type 2 DM
UAER: urinary albumin excretion rate
WMD: weighted mean difference
24-h UTP: 24-h urinary total protein
